# Comparing Palm Oil, Tocotrienol-Rich Fraction and α-Tocopherol Supplementation on the Antioxidant Levels of Older Adults

**DOI:** 10.3390/antiox7060074

**Published:** 2018-05-28

**Authors:** Nor Helwa Ezzah Nor Azman, Jo Aan Goon, Siti Madiani Abdul Ghani, Zalina Hamid, Wan Zurinah Wan Ngah

**Affiliations:** 1 Department of Biochemistry, Faculty of Medicine, Universiti Kebangsaan Malaysia, Jalan Yaacob Latif, Kuala Lumpur 56000, Malaysia; ezzahafezzi@gmail.com (N.H.E.N.A.); ctiemadiani@gmail.com (S.M.A.G.); wwanzurinah@yahoo.com (W.Z.W.N.); 2 Sime Darby Foods and Beverages Marketing Sdn. Bhd, Petaling Jaya 47301, Selangor, Malaysia; zalina.hamid@simedarby.com

**Keywords:** aging, oxidative stress, vitamin E, oxidative status, enzyme

## Abstract

Background: Tocotrienol and tocopherol are known to prevent numerous degenerative diseases. The aim of this study is to compare the effects of tocotrienol-rich fraction (TRF) with α-tocopherol (α-TF) on the antioxidant status of healthy individuals aged between 50 and 55 years. Methods: Volunteers were divided into groups receiving placebo (*n* = 23), α-TF (*n* = 24) and TRF (*n* = 24). Fasting venous blood samples were taken at baseline (0 month), 3 months and 6 months of supplementation for the determination of superoxide dismutase (SOD), catalase (CAT), and glutathione peroxidase (GPx) activities as well as for reduced glutathione (GSH) and oxidized glutathione (GSSG) concentrations. Results: CAT and GPx were unaffected by TRF and α-TF supplementations. SOD activity increased significantly after six months of TRF supplementation. Analysis by gender showed that only female subjects had significant increases in SOD and GPx activities after six months of TRF supplementation. GPx activity was also significantly higher in females compared to males after six months of TRF supplementation. The GSH/GSSG ratio increased significantly after six months of TRF and α-TF supplementation in only the female subjects. Conclusion: TRF and α-TF supplementation exhibited similar effects to the antioxidant levels of older adults with TRF having more significant effects in females.

## 1. Introduction

Vitamin E is a potent chain-breaking antioxidant that has been shown to reduce oxidative stress and prevent the propagation of free radical reactions. There are eight naturally occurring forms of vitamin E that are distinguished by the methylation pattern on the chromanol ring. These are further divided into four isomers designated as alpha (α), beta (β), delta (δ), and gamma (γ) tocopherols and tocotrienols [1]. Tocopherols and tocotrienols have the same basic chemical structure characterized by a long chain attached at the second position of a chromanol ring. Tocotrienols differ from tocopherols because they possess a farnesyl rather than a saturated isoprenoid C_16_ side chain [2]. All tocopherols and tocotrienols are potent antioxidants with lipoperoxyl radical-scavenging activities [3]. Based on a recent review, tocotrienol is not considered a part of the vitamin E family because it has distinct biological activities, cellular targets and molecular mechanism of action that is different from α-tocopherol [4]. In tabulating the Dietary Reference Intakes for vitamin E, the United States Pharmacopoeia also standardizes the units definition of different vitamin E analogues according to the efficiency of α-tocopherol because β-, γ- and δ-tocopherol and the tocotrienols are considered to be less active [5].

Most research on vitamin E has primarily focused on α-tocopherol (α-TF) [6] because α-TF is the predominant form of vitamin E in tissues and human plasma. However, varying outcomes have been obtained regarding its protective role in the prevention or treatment of chronic diseases such as cancer and cardiovascular diseases [7,8]. Tocotrienol has been reported to be more effective in the prevention of disease progression compared to tocopherol [9,10,11,12,13,14,15]. However, most studies were done using animals and in vitro models. To date, very limited data is available from human interventional studies using tocotrienol.

We previously reported that tocotrienol-rich fraction (TRF) supplementation restored the redox balance of individuals over 50 years of age after six months of supplementation [16,17]. Increases of plasma α-TF and tocotrienol with supplementation have been associated with a reduction in DNA damage, and improvements in lipid profile and oxidative status in older adults [17,18]. To the best of our knowledge, studies comparing the efficacy between tocopherols and tocotrienols supplementation in humans are still scarce and gender response disparities to the supplements is yet to be determined.

Humans are equipped with an extremely sophisticated and complex antioxidant protection system to protect the cells and organ systems of the body against free radicals [19,20]. The enzymatic components of the antioxidative defense system comprises several antioxidant enzymes such as superoxide dismutase (SOD), catalase (CAT), glutathione peroxidase (GPx), enzymes of ascorbate-glutathione (AsA-GSH) cycle, ascorbate peroxidase (APX), monodehydroascorbate reductase (MDHAR), dehydroascorbate reductase (DHAR), and glutathione reductase (GR) [21] as well as non-enzymatic antioxidant systems such as bilirubin, glutathione (GSH), carotenoids, and vitamins C and E. The antioxidant enzymes operate in different subcellular compartments and respond in concert when cells are exposed to oxidative stress [22]. However, the effectiveness of the natural antioxidant network has been reported to decline with aging [23,24,25]. Thus, the purpose of this study is to compare the effects of TRF and α-TF supplementation, respectively, on the antioxidant levels of healthy individuals aged between 50 and 55 years.

## 2. Materials and Methods

### 2.1. Study Subjects

The protocol of this study was approved by the Research and Ethics Committee of the Faculty of Medicine, Universiti Kebangsaan Malaysia (UKM 1.5.3.5/244/FF-434-2011). A total of 237 volunteers aged 50 to 55 years were approached for general health screening. Volunteers with a positive history of cardiovascular disease, stroke, chronic inflammatory diseases, hematological diseases or surgery within 6 months; serious medical illness such as cancer, malignant hypertension, uncontrolled diabetes or cardiopulmonary insufficiency; and consumption of supplements or pharmaceutical products with antioxidant properties were excluded. Physical activity of volunteers was not documented. However, volunteers were advised to be persistent with their daily physical activities when enrolled into the study to prevent modifications of oxidative status in the body by sudden onset of any exercise regime. A total of 86 healthy, non-smoking and non-alcoholic volunteers were enrolled into the study after obtaining written informed consent. Among this number, 71 subjects completed the supplementation study, while 15 subjects dropped out due to personal reasons and health problems.

### 2.2. Experimental Design

Recruited subjects were randomly divided into three groups and supplemented accordingly for 6 months with placebo (*n* = 23), α-TF (400 IU/day) (*n* = 24) and TRF (150 mg/day) (*n* = 24). The doses of supplementation used were based on positive results of previous human studies [26,27,28,29]. All subjects were requested to consume the supplements after dinner to ensure proper absorption [30,31] and encouraged to maintain their usual diet and lifestyle throughout the study period. The supplements including placebo were supplied by Sime Darby Food and Beverages Marketing Sdn. Bhd (previously known as Sime Darby Bioganic). Each TRF capsule contained 70.4 mg α-tocotrienol, 4.8 mg β-tocotrienol, 33.6 mg δ-tocotrienol, 57.6 mg γ-tocotrienol, and 48 mg α-tocopherol. α-TF capsules contained only tocopherol, while the placebo contained olive oil. The treatment was double-blinded throughout the study period until all data were collected, after which the randomization code was broken. Compliance of subjects was verified by the determination of plasma TRF and α-TF levels by high-performance liquid chromatography. The data of increased TRF and α-TF plasma levels after supplementation was reported earlier in a separate publication [23].

### 2.3. Sample Collection

Blood sampling was performed at baseline (0 months), 3 months and 6 months of supplementation. Venous blood samples (15 mL) were drawn into heparin tubes between 08:00 a.m. and 10:00 a.m. and processed within 1 to 2 h. Plasma was separated by centrifugation at 1800 rpm for 30 min at 4 °C. The obtained erythrocytes were washed two times with 0.9% sodium chloride solution. The mixture was then separated again by centrifugation at 3000 *g* for 10 min at 4 °C. Washed erythrocytes were used for the determination of antioxidant activities (SOD, CAT and GPx) and non-enzymatic antioxidant (reduced GSH and oxidized glutathione (GSSG)). Samples were then stored at −80 °C until further analysis.

### 2.4. Determination of Erythrocyte Antioxidant Enzymes (SOD, CAT, GPx)

Blood antioxidant enzymes activity (SOD, CAT and GPx) were measured via Cayman Chemical assay kit (Cayman Chemical Company, Ann Arbor, MI, USA). The SOD assay kit utilizes a tetrazolium salt for detection of the superoxide radicals generated by xanthine oxidase and hypoxanthine. The amount of enzyme required to exhibit 50% dismutation of superoxide radical is defined as one unit of SOD. This assay kit can measure three types of SOD: Cu/Zn, Mn, and FeSOD. SOD activity was expressed as U/mL.

The CAT assay kit is based on the reaction of the enzyme with methanol in the presence of an optimal concentration of hydrogen peroxide (H_2_O_2_). The formaldehyde produced was measured colorimetrically with Purpald, a chromogen that specifically forms a bicyclic heterocycle with aldehydes, which upon oxidation changes from colorless to a purple color and quantified at an absorbance of 540 nm. CAT activity was expressed as nmol/min/mL.

GPx activity was determined via the coupled reaction with GR and measured by a decreased absorbance at 340 nm. GPx produces GSSG upon reduction of hydroperoxide while GR returns GSSG to its reduced state. GPx activity was expressed as nmol/min/mL.

### 2.5. Determination of Erythrocyte GSH and GSSG Concentration

Reduced GSH and GSSG were measured by Cayman Chemical Glutathione assay kit (Cayman Chemical Company, Ann Arbor, MI, USA). The assay kit used glutathione reductase through the enzymatic recycling method for the quantification of GSH. The sulfhydryl group of GSH reacts with 5,5′-dithio-bis-2-nitrobenzoic acid (DTNB) to form a yellow-colored 5-thio-2-nitrobenzoic acid (TNB). The rate of TNB production was directly proportional to the concentration of GSH in all samples. The erythrocyte lysate was deproteinated using metaphosphoric acid (Sigma-Aldrich, St. Louis, MO, USA), 2-vinylpyridine (Merck, Jerman, Kenilworth, NJ, USA) and triethanolamine (Sigma-Aldrich, St. Louis, MO, USA) to avoid interferences from particulates and sulfhydryl group in proteins. GSH concentration in the samples was measured at 405–414 nm absorbance and expressed as µM.

### 2.6. Statistical Analysis

Statistical analysis was performed using the Statistical Package for Social Sciences (SPSS) Version 22.0 (IBM Corp., Chicago, IL, USA). The Shapiro–Wilk test was used to check the normality of the variables. Statistical evaluation was measured using two-way analysis of variance (ANOVA) test to compare changes from baseline to 3 and 6 months for all variables to verify the effects of treatment. A post hoc Least Significant Difference (LSD) multiple comparison test was performed to compare the average of the treatment group to the average of the control group and the impact of gender. Statistical tests with *p* < 0.05 were considered as significant. All the results are presented in mean ± S.E.M.

## 3. Results

### 3.1. Demography of Subjects

The mean ± SEM age for the subjects were 52.2 ± 2.1 for placebo, 52.5 ± 2.5 for α-TF, and 53.4 ± 1.5 for TRF. The number of subjects from each gender was comparable for the placebo, α-TF, and TRF groups (Table 1). The mean age of supplemented groups was not significantly different compared to placebo.

### 3.2. Antioxidant Enzymes Activities (SOD, CAT and GPx)

**Figure 1:**
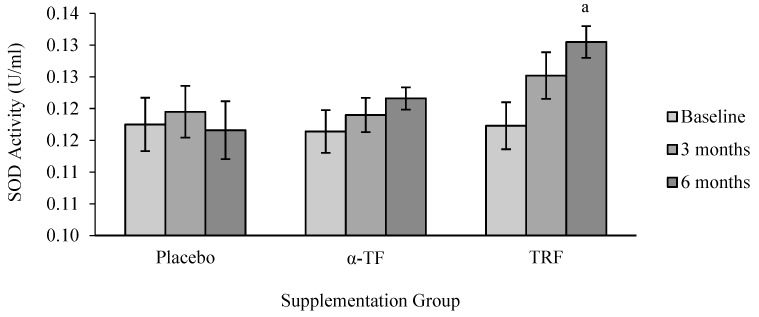
Effect of α-tocopherol (α-TF) and tocotrienol-rich fraction (TRF) supplementation on superoxide dismutase (SOD) activity for 6 months. a = statistically significant compared to 0-month TRF group, *p* < 0.05.

**Figure 2:**
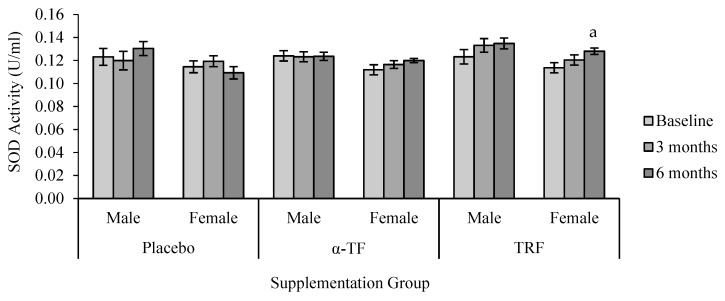
Effect of α-TF and TRF supplementation between males and females on SOD activity for 6 months. a = statistically significant compared to 0-month TRF group (female), *p* < 0.05.

A significant effect was observed in SOD activity with supplementation of TRF. TRF supplementation significantly increased SOD activity after 6 months of treatment compared to baseline (0 month) (Figure 1). Further analysis by grouping of gender showed that SOD activity increased in female subjects after 6 months of TRF supplementation compared to baseline (0 month) (Figure 2).

**Figure 3:**
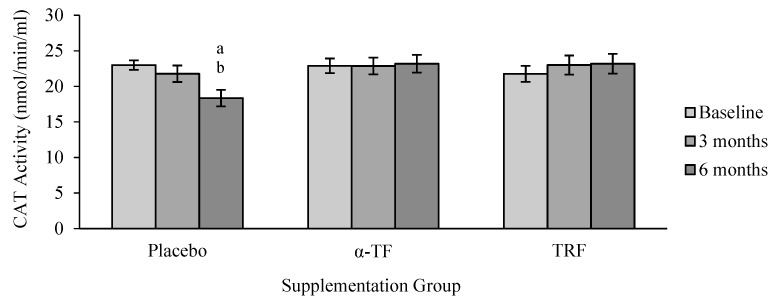
Effect of α-TF and TRF supplementation on catalase (CAT) activity for 6 months. a = statistically significant compared to 0-month placebo group, b = statistically significant compared to 3 months placebo group, *p* < 0.05.

**Figure 4:**
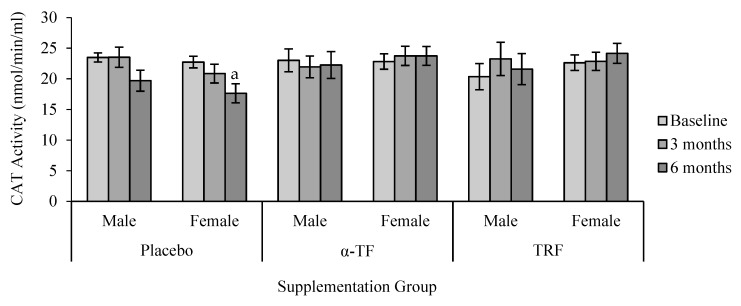
Effect of α-TF and TRF supplementation between males and females on CAT activity for 6 months. a = statistically significant compared to 0-month placebo group (female), *p* < 0.05.

CAT activity remained unchanged in TRF and α-TF supplemented groups (Figure 3). By contrast, CAT activity decreased after 6 months in the placebo group, particularly among the female subjects when compared to the baseline (0 month) (Figure 4).

**Figure 5:**
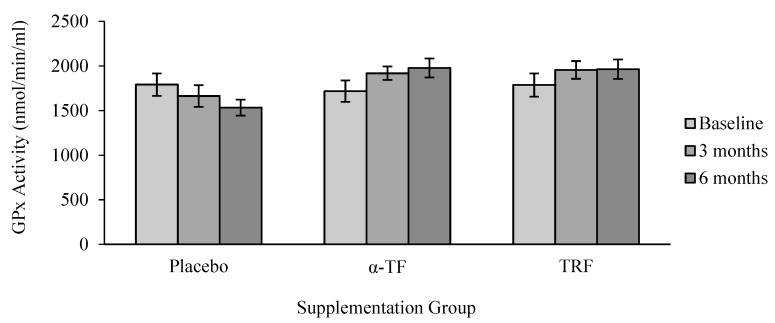
Effect of α-TF and TRF supplementation on glutathione peroxidase (GPx) activity for 6 months. GPx activity remained unchanged throughout the study with both supplements.

**Figure 6:**
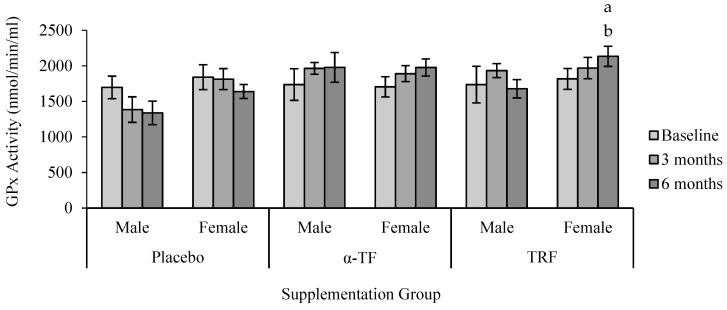
Effect of α-TF and TRF supplementation between males and females on GPx activity for 6 months. a = statistically significant compared to 0-month TRF group (female), b = statistically significant compared to 6 months TRF group (male), *p* < 0.05.

However, no significant differences were found in the activity of GPx between all groups (Figure 5). Similar to the results of SOD, only female subjects had increased GPx activity after 6 months of TRF supplementation compared to baseline (0 month) (Figure 6).

### 3.3. Non-Enzymatic Antioxidant (GSH and GSSG)

**Figure 7:**
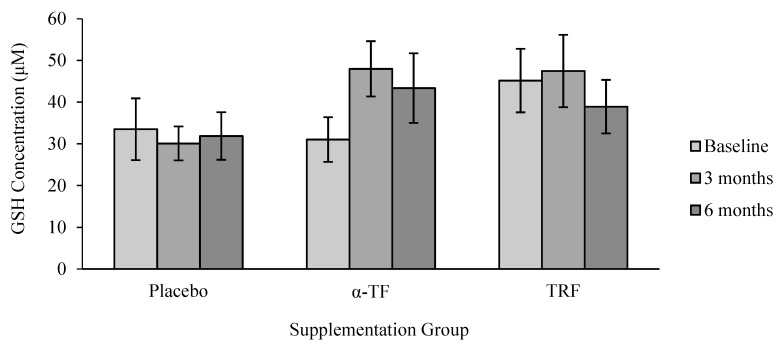
Effect of α-TF and TRF supplementation on glutathione (GSH) concentration for 6 months. The level of GSH remained unchanged throughout the supplementation period in both groups of vitamins.

**Figure 8:**
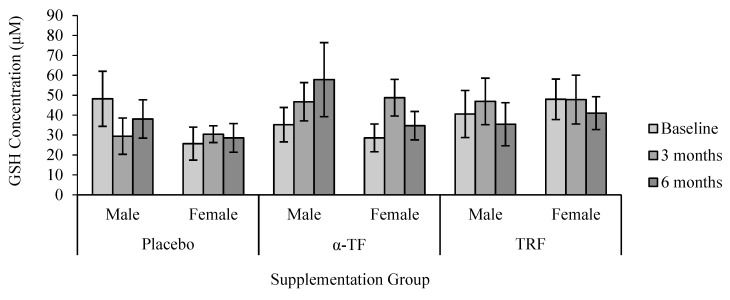
Effect of α-TF and TRF supplementation between males and females on GSH concentration for 6 months. No differences in the level of GSH were found between genders in all supplemented groups.

No significant differences were found in the concentration of GSH with TRF and α-TF supplementations (Figure 7). In addition, there were no apparent differences in GSH concentration between male and female subjects after TRF and α-TF supplementation (Figure 8).

**Figure 9:**
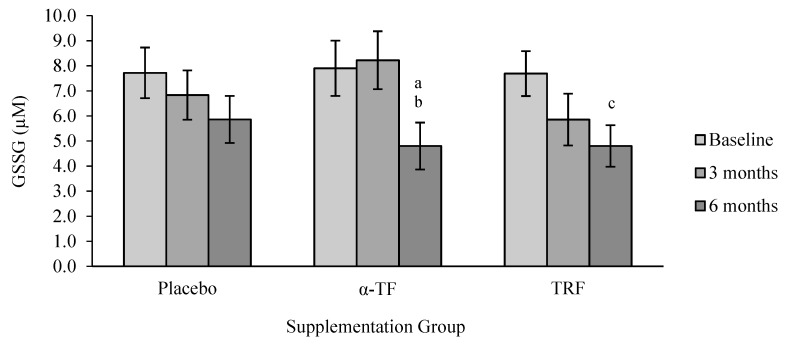
Effect of α-TF and TRF supplementation on oxidized glutathione (GSSG) concentration for 6 months. a = significantly different compared to 0-month α-TF, b = significantly different compared to 3 months α-TF, c = significantly different compared to 0 month TRF, *p* < 0.05.

**Figure 10:**
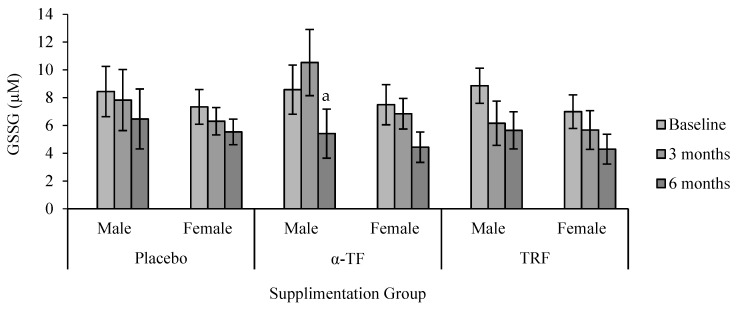
Effect of α-TF and TRF supplementation between males and females on GSSG concentration for 6 months. a = significantly different compared to 3-month α-TF (male), *p* < 0.05.

GSSG concentration was significantly decreased after 6 months of α-TF supplementation compared to baseline (month 0) and 3 months (Figure 9). Similarly, TRF supplementation also decreased the concentration of GSSG after 6 months of supplementation. Analysis by gender revealed a marked reduction of GSSG in the male subjects after 6 months of α-TF supplementation (Figure 10). No differences were observed between the two genders in the TRF and placebo groups.

**Figure 11:**
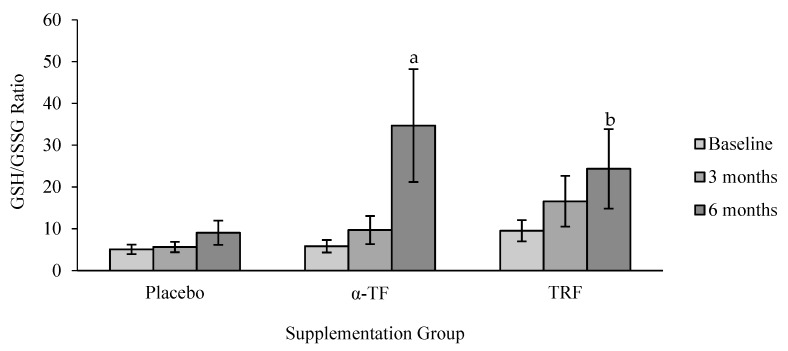
Effect of α-TF and TRF supplementation on GSH/GSSG ratio for 6 months. a = significantly different compared to 0-month α-TF, b = significantly different compared to 0-month TRF, *p* < 0.05.

**Figure 12:**
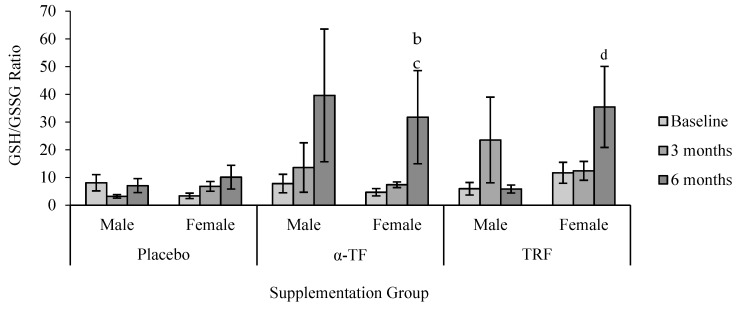
Effect of α-TF and TRF supplementation between male and female on GSH/GSSG ratio for 6 months. b = significantly different compared to 0-month α-TF (female), c = significantly different compared to 3-month α-TF (female), d = significantly different compared to 0-month TRF (female), *p* < 0.05.

The ratio of GSH/GSSG increased after 6 months of TRF and α-TF supplementation (Figure 11). These effects were seen only in female subjects with both supplementations (Figure 12).

## 4. Discussion

Oxidative stress is known to increase with age. Many studies have highlighted the benefits of vitamin E as an antioxidant to prevent damages to macromolecules in aging [27,32]. Results of this comparative study between TRF and α-TF further elucidate the role of these vitamin E isomers in preventing oxidative damages as reported previously.

SOD is an intracellular enzymatic antioxidant that catalyzes the conversion of superoxide anions to molecular oxygen and H_2_O_2_, whereas CAT and GPx promote the conversion of H_2_O_2_ into water and oxygen [33]. In this study, the activities of SOD and GPx were found to increase after 6 months of TRF supplementation. These outcomes are seen to be beneficial as SOD and GPx help neutralize reactive oxygen species that are generated extensively with age.

The activity of CAT remained unchanged throughout the study period regardless of supplementation with TRF or α-TF. However, its activity decreased after six months in the placebo group. Given the fact that both CAT and GPx remove H_2_O_2_ in the antioxidant network, the reduction of CAT activity suggests that the removal of H_2_O_2_ was predominantly accomplished by GPx in the placebo group. In line with the current results, TRF supplementation was also found to increase the activity of GPx without altering the activity of CAT in our previous study [16].

The unaltered activity of CAT with TRF and α-TF observed in this study is seen as a positive effect because the placebo group had decreased CAT activity after six months of study. Both supplements may have helped to maintain the oxidative status of the older adults through upregulation of other antioxidants as reported earlier.

According to a previous finding, premenopausal women aged 53.2 ± 5.7 years have been found to have increased oxidative stress manifested by elevated total antioxidant status [34]. The development of oxidative stress was suggested to be a consequence of reduced levels of circulating estrogens and increased abdominal and total body fat in women. Although it was not ascertained whether the female subjects in the present study were premenopausal, TRF supplementation effectively increased SOD and GPx activities in females of similar age.

The discrepancy between the response to antioxidant supplementation seen between men and women in this study could be due to the difference in the level of estrogen. This hormone has been implicated as a phenolic antioxidant due to the presence of phenolic A-ring in its structure [35]. Previous researches have shown that estrogen can upregulate the expression of antioxidant enzymes and protect against the release of cytochrome c by mitochondria [36,37]. This hormone is known to decrease in aged women but not in aged men [38]. We postulate that the level of estrogen in the female subjects of this study could have been lower compared to men, hence their antioxidant status was more receptive and responsive to antioxidant therapy. However, further research is needed to determine the extent of the role of estrogen in the oxidative status of women compared to men in aging.

Glutathione is an important constituent in the endogenous antioxidant defense system. Reduced GSH is a biological antioxidant that helps to protect the cells from reactive oxygen species such as free radicals, heavy metals, lipid peroxides and peroxides. It is the major endogenous antioxidant produced by the cells, participating directly in the neutralization of free radicals and reactive oxygen compounds, as well as maintaining exogenous antioxidants [39,40]. GSH is synthesized in the liver and GSH deficiency is associated with aging, cardiovascular disease, and cancer [41,42,43,44].

GSSG is usually reduced into reduced GSH via GSSG reductase, an enzyme that uses nicotine adenine dinucleotide phosphate (NADPH) as a cofactor [45]. The increase in GSSG levels is associated with oxidative stress [46,47] and may contribute to many chronic degenerative diseases. High level of GSSG is also found to promote the formation of extensive disulfide mixture and deactivation of SH-dependent enzyme.

The ratio of reduced GSH to GSSG levels in the blood is an indicator of cellular health. The GSH/GSSG ratio is considered very important as an index of oxidative status and guidance to a range of chronic risk to humans [48,49]. Decreased ratio of GSH/GSSG was proposed as a major contributing factor to some chronic diseases such as pneumonia, amyotrophic lateral sclerosis, kidney failure, malignant disorders, diabetes, high blood pressure, Parkinson’s disease, Alzheimer’s disease, and cataract formation [50,51,52,53,54].

In this study, supplementation of TRF and α-TF significantly increased the ratio of GSH/GSSG after 6 months. Tocotrienol and tocopherol are known to result in noncompetitive inhibition toward the substrates (GSH and 1-chloro-2,4-dinitrobenzene) of glutathione S-transferase (GST). Conformational changes of the enzyme lead to marked reduction of GST activity [55]. The mechanistic action of TRF and α-TF towards GST together with an improved redox balance could have led to the increase of GSH and reduction of GSSG concentrations as measured in this study after six months of supplementation. Interestingly, α-TF and TRF supplementations increased the ratio of GSH/GSSG only in the female subjects. Since not many studies have compared the levels of GSH and GSSG between genders in humans, further research engaging a larger number of subjects is needed to elucidate gender-associated differences in oxidative status.

## 5. Conclusions

TRF supplementation showed comparable effects to α-TF in modulating the activities of antioxidant enzymes and increasing the ratio of GSH/GSSG in healthy older adults. TRF seemed to improve the antioxidant levels of females more significantly compared to males. Nevertheless, a study with a bigger sample size is necessary to conclude this gender-associated disparity as this study is limited to an unequal number of males and females in every supplemented group. 

## Figures and Tables

**Table 1:** Baseline and intervention characteristics of the subjects.

	Group								
	Placebo			α-Tocopherol			(Tocotrienol-Rich Fraction) TRF		
	Male	Female	Total	Male	Female	Total	Male	Female	Total
Subjects (*n*)	8	15	23	9	15	24	9	15	24
Age (years)	52.7 ± 1.6	52.1 ± 1.7	52.2 ± 2.1	53.5 ± 1.5	52.7 ± 1.2	52.5 ± 2.5	53.1 ± 1.3	52.7 ± 1.9	53.4 ± 1.5
*p* value	-	-	-	0.335	0.49	0.845	0.609	0.678	0.935
